# Influence of inventory management practices on the availability of emergency obstetric drugs in Rwandan public hospitals: a case of Rwanda Southern Province

**DOI:** 10.1186/s12913-023-10459-x

**Published:** 2024-01-04

**Authors:** Jean Claude Kabera, Marie Françoise Mukanyangezi

**Affiliations:** 1 EAC Regional Centre of Excellence for Vaccines, Immunization, and Health Supply Chain Management, College of Medicines and Health Sciences, University of Rwanda, Kigali, Rwanda; 2 Department of Pharmacy, School of Medicine and Pharmacy, College of Medicine and Health Sciences, University of Rwanda, Kigali, Rwanda

**Keywords:** Inventory management, Emergency obstetric drugs, Logistic management tools, Availability, Public hospitals, Rwanda

## Abstract

**Background:**

Stock-outs of some life-saving drugs, such as emergency obstetric drugs, are evident in many health facilities and have been reported to be the leading cause of maternal mortality and morbidity for women from low and middle income countries (LMICs). For many cases, this situation is associated with poor inventory management practices. The aim of this study was to investigate the influence of inventory management practices on the availability of emergency obstetric drugs in Rwandan public hospitals: case of the Rwanda Southern Province. Moreover, to gain a better grasp of the problem and to suggest possible areas for improvement.

**Methods:**

An institutional-based cross-sectional study was carried out in all ten district hospitals (DHs) providing maternal health care and dispensing emergency obstetric drugs namely; Kigeme DH, Munini DH, Kabutare DH, Kibilizi DH, Gakoma DH, Nyanza DH, Ruhango DH, Gitwe DH, Kabgayi DH and Remera Rukoma DH. Both quantitative and qualitative data were collected and analyzed. Oxytocin injection, Misoprostol tablet and Magnesium sulphate injection as recommended emergency obstetric drugs by WHO, UNFPA and Rwanda Essential Medicines list were included in the study.

**Results:**

The study revealed that keeping logistics management tools up to date is the backbone of inventory management practices in the availability of medicines and medical supplies. The results showed that hospitals with up-to-date logistics tools for their pharmaceutical management were 33.25 times more likely to have their emergency obstetric drugs in stock at all times compared to those that do not regularly update their logistics tools. The proper use of bin cards and electronic software (e-LMIS) contributed greatly to reducing the stock-out rate of emergency obstetric drugs by 89.9% and reduction of unusable to usable stock ratio by appropriate use of simple techniques such as the Min–Max inventory control model by 79%. Over an 18-month period, misoprostol tablet had the highest average days (32) of stock-outs (5.9%), followed by magnesium sulphate injection with an average of 31 days (5.7%), and oxytocin injection with an average of 13 days (2.4%).

**Conclusion:**

Proper use of pharmaceutical management tools within hospitals premises positively influence the availability of life-saving drugs, such as emergency obstetric drugs. Adequate supply chain staffing in health facilities is the most important key to improving inventory management practices and medicine availability.

## Background

The five most important direct causes of maternal mortality and complications in LMICs are hemorrhage, sepsis, unsafe abortion, eclampsia, and obstructed labor [1]. Postpartum Hemorrhage (PPH) and Pre-Eclampsia/Eclampsia (PE/E) are the two leading causes of death accounting for almost half of maternal deaths in Sub-Saharan Africa (SSA) [2]. In almost all cases, these conditions are preventable or treatable, and most important intervention in reducing maternal mortality and morbidity is to ensure that the emergency obstetric drugs and related medical supplies are consistently available in the health facilities [3]. The first measure taken to stop bleeding is the use of drugs [2]. Affordable and effective medicines are available to treat PPH and PE/E and prevent maternal deaths. Three low-cost drugs (Oxytocin injection, Misoprostol tablets and Magnesium Sulfate injection), if made widely available, could save many of the lives of women who die each year [4].

In public health facilities, a lack of essential medical supplies hinders an effective maternal healthcare service delivery. The situation is particularly evident in SSA countries [5]. It is estimated that 30% of the world's population lacks essential medicines they need to manage several conditions [6]. The causes of shortage are multifactorial and include supply, demand, and regulatory issues. Supply issues consist of manufacturing problems, unavailability of raw materials, logistic problems, and business problems [7].

In Rwanda, a recent study conducted in public health facilities of the Northern province of Rwanda revealed that 73% of health facilities faced a challenge of medium levels of stock outs (> 5–10% of drugs stocked out per month) to high levels of stock-outs (> 10% of drugs stocked out per month) which was partly attributed to the limited capacity of staff managing logistics and supplies at health facilities [8]. It has been shown that inadequate supply of drugs and improper management were identified among the main obstacles in providing 24-h quality Emergency Obstetric Care (EmOC) services, especially in remote and rural areas [5].

It is evidenced that proper inventory management practices are required to avert the frequent stock-outs of drugs, particularly in public health institutions [9]. Pharmaceutical inventory management is a complex, but vital process within the healthcare delivery system. It is a systematic approach to sourcing, receiving, storing, issuing, and replenishing a given group of health products [10]. Poor inventory management might lead to overstocking or understocking of essential medicines leading to resource wastage and increased morbidity and mortality [11].

The concept of EmOC is based on the assumption that maternal complications are unpredictable, and that obstetric complications can occur in 15% of deliveries [12]. When these difficulties arise, maternal mortality could be avoided in an environment where skilled health personnel, drugs and medical supplies necessary for EmOC are available [2]. Despite its importance in the provision of quality EmOC services, policymakers or researchers often fail to mention shortages of drugs and medical supplies when addressing the causes of poor maternal health services [13].

In LMICs, many studies focused mostly on assessing drug availability of program products for malaria, HIV/AIDS, and tuberculosis, and few studies have addressed the drug shortages in the provision of maternal health care services [12–14]. Effective pharmaceutical supply system maintains a steady supply to patients while minimizing the costs of holding inventory of medical products [15] but little is known on the challenges and extent to which pharmaceutical inventory management practices influence the availability of emergency obstetric drugs in Rwandan public hospitals. Therefore, this study assessed the inventory management practices used for the above mentioned emergency obstetric drugs in public hospitals of the Rwanda Southern Province.

## Materials and methods

### Study settings

The study was conducted in the ten district hospitals (DHs)) namely; Kigeme DH, Munini DH, Kabutare DH, Kibilizi DH, Gakoma DH, Nyanza DH, Ruhango DH, Gitwe DH, Kabgayi DH and Remera Rukoma DH of Rwanda Southern Province. The latter has approximately 5,963 km^2^ in area, borders Burundi in the South and has eight Districts. In Rwandan health system, each District has at least one district hospital. This hospital treats mainly the patients referred from health centers within district boundaries. At this level of healthcare service, two or more general medical doctors are available while lower levels of health centers are managed by nurses. Every month, DH procures medicines from District branch of Rwanda Medical Supply (RMS) limited. Hospital pharmacist receives medicines and medical supplies in central pharmacy and distributes them in different hospital subunits including maternity ward.

Inclusion criteria were presence of main hospital pharmacy and maternity unit and Emergency obstetric drugs while other medical supplies were excluded from the study.

### Study design and population

Institutional cross sectional-based study was conducted in the ten public hospitals in Rwanda, Southern Province from July 2020 to December 2021. Both quantitative and qualitative date were collected. All district hospitals in Rwanda South province were included in this study. Three EmObs (Oxytocin injection, Misoprostol tablet and Magnesium sulphate injection), representatives of EmObs drugs expected to be found in DHs, were used to assess availability of Emergency obstetric drugs at the day of survey and during the previous 18 months from June 2020 to December 2021. The target population to participate in this study were four staff at each hospital, three clinical staff (Medical doctor, Pharmacist, and midwife) and finance manager. The above people play major role in rational use and availability of emergency obstetric drugs. This make up a total of 40 staff as respondents.

### Data collection

Both quantitative and qualitative data were collected in this study. The instruments used in this research was a structured questionnaire that helped to identify inventory management practices in use, interview guides that helped to collect views and attitudes on the challenges affecting inventory management practices and availability of emergency obstetric drugs, and a checklist used to gather data on the availability status of EmObs drugs. A pilot study was carried out in Masaka and Kibagabaga DHs for collection tools validation.

The absence or presence of an item was shown by marking 'yes' or 'no'. The checklist ensured that all aspects were considered in totality. Quantitative data were also collected on explanatory variables including sociodemographic of health professionals, use of stock keeping tools (bin cards and e-LMIS), source of drug supply, availability of emergency obstetric drugs, the presence of inventory management practices. The historical logistics data were collected from e-LMIS and bin cards.

The collection of quantitative data was done by the principal investigator using the "drop off and pick up later" technique. Qualitative data were collected through an in-depth interview by audio recording that could take around twenty minutes with each participant. Key informant interview was carried-out to provide in-depth information on the inventory management practices and availability of Emergency Obstetric medicines in public hospitals in Rwanda Southern Province.

### Data analysis

Quantitative data generated from the questionnaires and checklist were validated to check for completeness, coded and fed into STATA 13. Univariate, bivariate and logistics regression analysis were done. The data collected were analyzed by both qualitative and quantitative methods. Descriptive statistics (frequency distribution and percentages) were computed. Chi-square test was used to test for the association between variables. Analyzed quantitative data were then presented using tables and figures. Thematic method was used to analyze qualitative data. Data were tape recorded, translated, transcribed and categorized into themes.

## Results

The total of ten hospitals dispensing emergency obstetric drugs in Southern province, Rwanda were included in this study. Out of 40 personnel expected only 38 accepted and participated in this study.

### Demographic characteristics of study participants

Of the 38 participants, 22 (57.89%) and 16 (42.1%) were males and females, respectively. Also, 18 (47.4%) participants were between 40–50 years of age. The role of supply chain management of emergency obstetric drugs was assigned to pharmacists in 8 (80%) hospitals, and nurse store managers in 2 (20%) hospitals. Majority of respondents (71%) were working in the hospitals for a period between 1–5 years (Table 1).

**Table 1:** Characteristics of study participant (*n* = 38)

Variable	Staff/interval	Frequency (%)
**Type of personnel**	Pharmacists	8 (21%)
	Nurses store managers	2(5.2%)
	Medical Doctors	10 (26.31%)
	Midwives	10 (26.31%)
	Administrators	8 (21%)
**Sex**	Male	22 (57.89%)
	Female	16 (42.1%)
**Age (years)**	Below 30 years	0 (0%)
	30 – 40	12 (31.57%)
	40 – 50	18 (47.36%)
	Above 50	8 (21%)
**Work experience (years)**	Below 1 year	0 (0%)
	1 – 5	27 (71%)
	5 – 10	9 (23.68%)
	Above 10	2 (5.26%)

### Inventory management practices in different hospitals of the Southern Province in Rwanda

This study showed that inventory management practices available in all hospitals were accurately completeness of inventory management tools, stock monitoring using Min–Max inventory control system; determining their own needs of emergency obstetric drugs using formula based on the past consumption patterns with current stock on hand and periodical physical inventory count. Accuracy of stock records balance ranged from 49.5% to 100% while stock record balance that were higher than physical inventory ranged from 0 to 23.3%.

**Fig. 1:**
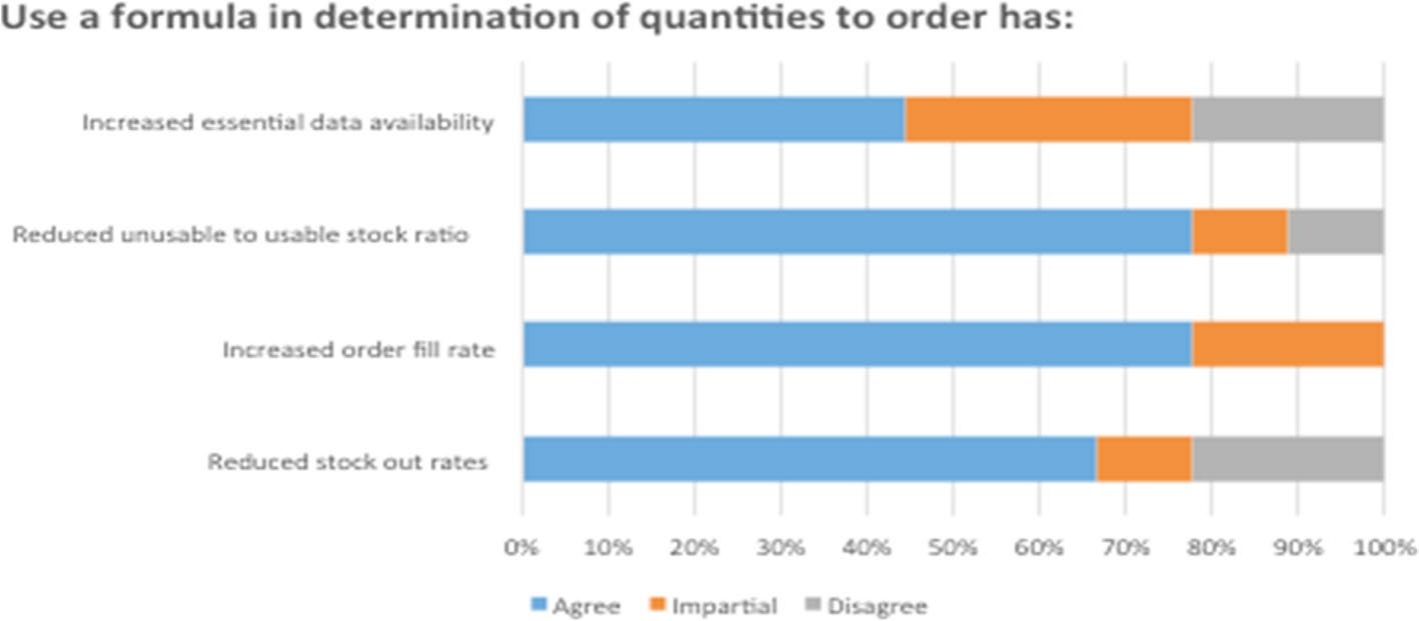
Outcomes of using Min–Max inventory control formula in the process of medical drugs’ ordering

The findings showed that the most predominantly type inventory control model used was forced ordering Max–Min inventory control system in 70% of hospitals. The 85% of respondents agreed that the use of above inventory management practices has reduced the stock out rate of emergency obstetric drugs and unusable to usable stock ratio at 79% (Fig. 1). Physical inventory count was conducted on a monthly basis in the all hospitals in the Rwanda Southern Province.

### Logistic tools in use

**Fig. 2:**
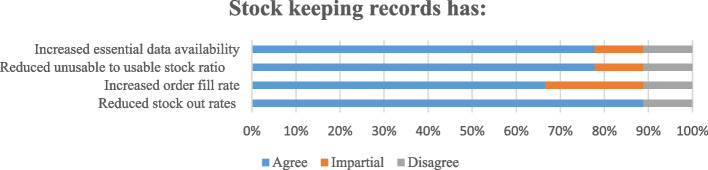
Outcomes of compliance in updating stock keeping records

Logistics management tools that hospitals used to manage health products include manual logistic tools and electronic software (Table 2). The manual logistics tools were stock cards, daily consumption registers, and internal requisition forms. The electronic software was electronic management information system (e-LMIS). Both electronic system and manual logistics tools for emergency obstetric drugs were present at 9 (90.0%) of all hospitals (Table 2). Also, 26 (86.6%) bin cards out of 30 in the 10 hospitals complied with the use of logistics tools of oxytocin injection, Misoprostol tablets and magnesium sulfate injection stocked. In assessing average stock out indicator for those three drugs in all ten district hospitals (in total 30 items), 11 (36.6%) had stock-out on the logistic tools within the last 3 months while 7 items (23.4%) had stock-out at the day of visit in the different hospitals. Furthermore, the proper use of bin cards and e-LMIS contributed greatly to reducing the stock-out rate of emergency obstetric drugs by 89.9% (Fig. 2).

**Table 2:** Logistics management tools hospitals used to manage health products

Characteristic of logistic tools	Frequency and percentage of hospitals (n)
*Types of a logistics tool for EmOBs (n* = *10)*	
Manual logistic tools only	0 (0.0%)
Electronic only	1(10.0%)
Both system	9 (90.0%)
Compliance on the logistics tool (for 3 EmOb drugs stored in 10 hospitals) *N* = 30	26 (86.6%)
*Stock out information*	
Stock-out on the logistic tool in the last 3 months (*N* = 30)	11(36.6%)
Stock-out on the day of visit (*N* = 30)	7 (23.3%)

### Stock levels of emergency obstetric drugs in the public hospitals of the Rwanda Southern Province

We studied the availability of emergency obstetric drugs in terms of order fill rate and lead time, availability, stock levels and wastage rate. All emergency obstetric drugs were ordered and supplied from Rwanda Medical Supply Limited (RMS Ltd) branches. We accessed the two last orders placed by each hospital and to what percentage it was served. A total of 20 orders were placed by concerned hospital. Of the 20 orders, only 11 (55.0%) were fully received whereas 7 (35.0%) were partially received, and 2 (10.0%) were not served. The duration for ordered emergency obstetric drugs to reach the facility was three days for 9 (50%) orders, seven days for 5 (27.7%) orders, two days for 2 (11.1%) orders, and only one day for 2 (11.1%) orders (Table 3).

**Table 3:** Hospitals' emergency obstetric drugs order fill rate

Features of sample	Percentage of two last orders processed (= 20)
**Order fill rate**	
Fully order received	11(55.0%)
Partially order received	7(35.0%)
Order not served	2(10.0%)
**Lead time**	
Seven days and above	5(27.7%)
Three days	9(50%)
Two days	2(11.1%)
One day	2(11.1%)

### Availability and stock levels of emergency obstetric drugs

The results showed that over the last 18 months and on the day of the visit, each of the four hospitals (40%) experienced at least once the stock out of oxytocin injection and the days of stock out ranged between 6–35 with average of 13 days, representing 2.4% in 18 months ago. Magnesium sulfate injection fell into stock out in 7 (70%) of hospitals, and the days of stock out ranged between 16–96 with an average of 31 days, corresponding to 5.7%. Misoprostol tablets felt into stock out in 3 (30%) hospitals and days of stock out ranged between 12- 95 with average of 32 days corresponding to 5.9% within last 18 months.

### Stock wastage rate of emergency obstetric drugs

Stock wastage rate was evaluated for all participating hospitals by comparing the unusable stock (damaged and expired) of emergency obstetric drugs to the total stock on hand (usable and unusable). No unusable stock of oxytocin injection was found in all hospitals which means that no stock wastage. Average stock wastage for misoprostol tablets and magnesium sulfate injection in all hospitals were 9.5% and 17.1%, respectively.

### Relationship of inventory management practices and availability of emergency obstetric drugs

To find influence inventory management practices have on the availability of emergency obstetric drugs, we used logistic regression analysis to identify how a specific practice can cause variation in the availability of those drugs (Table 4). A significant prediction of emergency obstetric drugs availability was found only with logistic tools updated or not updated (Coef: 20.6, *P*-value: 0.000).

**Table 4:** logistics tools updated and drug availability status

Logistics tool updated (yes/no)	Drug availability		Total
	0	1	
0	7	2	9
1	2	19	21
Total	9	21	30

The Pearson chi-square = 13.9758 with *p*-value = 0.000 Yes = 1 and No = 0. The X2 critical value for the significance test at α = 0.05 and df = 1 is 3.841. The calculated Χ^2^ = 13.97is greater than tabulated Χ^2^ = 3.841. Hence, there is significant relationship between current inventory management practices and the availability of emergency obstetric drugs in Rwanda's public hospitals, South Province.

The study showed that logistics tools updated was significantly associated with variable *“Drugs availability in the hospitals”* when *p*-value = 0.000 < 0.05, the Null hypothesis is rejected and conclude that there is significant relationship between current inventory management practices and the availability of emergency obstetric drugs in Rwanda's public hospitals, South Province.

Attempt of assessing the extent to which logistics tool updated would vary along the outcome variable *“Drugs availability in the hospitals”*, the analysis showed that 62.05% of the variations in obstetrics drugs availability would be as a result of its dependence on logistic tool updated.

The odds ratio showed that hospitals updated logistics tools for their health facilities were 33.25 times more likely to have their *obstetric* drugs in stock all the time as compared to those that don’t update their stock updated in their logistic tool (Table 5). Logistic tool updated refer to have all stock indicator as it is used in determining the quantity of medicine to be procured or validated.

**Table 5:** Variables: logistics tools updated and drug availability status

Logistics tool updated (yes/no)	Odds Ratio	Std. Err	P-value	[95% Conf. Interval]
Drug Availability	33.25	36.355	0.001	[3.900331, 283.4535]
Cons	0.2857	0.2291	0.118	[0.0593543,1.375345]

### Qualitative results

In addition to quantitative findings, a qualitative component of the research seemed to be best suited to uncover views in inventory management practices and EmOb drugs availability. Therefore, an interview guide was conducted. The themes being researched were: possible causes of inappropriate inventory management practices within hospitals, challenges encountered in managing emergency obstetric drugs and suggestions or recommendations to be formulated.

Inadequate staffing in the hospital pharmacy was highlighted as main cause of inaccurate stock records. A participant reiterated “the *problem is insufficient staff; I am the only pharmacist here, apart from supply chain activities, I am requested to participate in more than 4 other committees, such as Drug Therapeutic Committee (DTC), tender committee, Accreditation committee, and Infection Prevention and Control (IPC) committee. In addition, there is a high turnover of nurses that work in hospital pharmacy and the new comers are not familiar with completing logistic tools accurately in the beginning”.*

About challenges affecting the inventory management practices of emergency obstetric drugs, double work of using concomitantly the electronic and hard logistic tools, poor storage infrastructure and unpredictable or fluctuation in consumption of some products have been mentioned as main challenges in addition to unreliable supply of emergency obstetric drugs. A pharmacist has argued that “*first*, *a double work of using hard copies and e-LMIS because auditors and hospital administration are sticking on having hard copies in addition to the use of e-LMIS in inventory management. Secondly, some products are difficult to manage. For example, magnesium sulfate injection can become expired or stock out because of unpredictable consumption; and we experience difficulties in medicines arrangement, discrepancies during physical count caused by limited storage space and poor pharmacy storage infrastructure.*

Lack of commitment of top management to understand the workload of hospital pharmacy activities and needs was also highlighted by most of participants as challenge in performance of inventory management within hospital pharmacy.

Adequate staffing and awareness of hospital top management are main recommendations that emerged from this study as key interventions to improve inventory management practices of medicines within hospital pharmacy. *Participant highlighted that “availing at least four trained pharmacy professionals in the health supply chain management at district hospital may improve inventory management practices, thus ensuring continuous provision of all necessary medical supplies”.*

## Discussions

This study aimed to evaluate the influence of inventory management practices on availability of emergency obstetric drugs in the public hospitals of Rwanda, Southern Province.

The results of the study revealed that the most important inventory management practices used in management of emergency obstetric drugs in hospitals were accurate completeness of stock keeping logistical tools, stock monitoring using Min–Max inventory control system, using formula based on the past consumption patterns and stock on hand to determine the quantity to order and physical inventory count on the monthly basis. Assessed hospitals were found to use bin cards and electronic inventory management software as inventory management tools. Oxytocin injection was the drug with the fewest days of stock-outs, while magnesium sulphate injection faced the most days of stock-outs and overstocking in most public hospitals of Rwanda Southern Province.

In this study, the most predominant inventory management techniques practiced in the hospitals were Max–Min inventory control system in the stock monitoring practice, using past consumption patterns as a formula and keeping accurate and updated logistic tools. This finding is consistent with the other studies done in Nyamira and Meru Counties, Kenya [10, 16]. These results also corroborate with other study done in the Wollo university and found that minimum–maximum and consumption based are two simple and useful formulas to determine quantity to order when compared to more complicated mathematical formulas, such as economic order quantity, exponential smoothing demand [17]. This consistency could be attributed to the similarities of health systems between Rwanda and Kenya. However, the compliance with these practices should be slightly higher in Rwanda, as it is a landlocked country where almost all medical supplies are imported and a scheduled inventory control may be adequate.

In the current study, all public hospitals in the Southern Province of Rwanda were found to use stock cards and electronic inventory management software as main health supply chain tools and accuracy of completing them played a major role in ensuring the good stock levels of emergency obstetric drugs within hospitals. These results reflect those of a study conducted in Uganda where it was also found that having accurate stock cards available for each stock keeping unit was very important in determining stock levels and status [18]. The presence and correct use of stock-keeping tools is very important in the performance of the health supply chain management, as it is the source of data for procurement, service delivery monitoring and decision making [19].

The study unveiled that during the previous18 months from the visit, oxytocin injection experienced the fewest days of stock out when compared to misoprostol tablets and magnesium sulfate injection. This finding is similar to the one from a study conducted in the Malawi where oxytocin injection was available in most of health facilities [20]. However, magnesium sulfate injection was found to have more days of both stock out and overstock. The above scenario can be explained by the fact that healthcare providers are reluctant to use magnesium sulfate injection due to its complexity of administration and unwarranted fear of adverse effects as previously reported in North Karnataka, India [12, 21]*.* Low demand and use among healthcare providers can have a more systemic impact on its stock levels [22].

The status of overstocking of some emergency obstetric medicines found in this study must be placed in the context of the Rwandan health system and the organization of its health supply chain management. Rwanda Medical Supply Ltd is the largest supplier of emergency obstetric drugs to public health facilities [23, 24]. However, same EmObs drugs may be supplied under two different budget holders, one in the Maternal, Child and Community program (MCCH) and delivered to public health facilities free of charge, while other batches are to be purchased and sold to customers and may lead to expiries. Both stock-outs and overstocking have a negative impact on the healthcare system, as the former can lead to loss of life due to PPH and pre-eclampsia, while the latter can lead to expiry and related disposal costs [23, 25].

The current study found that inadequate staff was the main challenge in the pharmaceutical inventory management, the same issue has been identified in the study done in the Tanzania, on the medicine stock out and inventory management problems in the public hospitals [15, 26].

This study has a number of limitations; therefore, the results should be interpreted with caution. Firstly, the responses are self-reported and this is susceptible to social desirability bias. Secondly, the study was conducted in one Province located of Rwanda and the results may not be generalized to entire Country in the public hospitals. Despite the limitations, the study provides an insight into the inventory management practices in the public hospitals of Rwanda. Another limitation is that the study did not put much emphasis on EmObs drugs supplier performance.

## Conclusion

This research presents a detailed influence of inventory management practices on the availability of emergency obstetric drugs in the public hospitals of Southern province, Rwanda. The findings showed that the use Max–Min inventory system control in stock monitoring, use of consumption patterns and stock on hand in the formula of determining quantity to order and use of stock keeping logistic tools appropriately were main inventory management practices. Keeping logistic tool updated is a vital practice for inventory management performance. Oxytocin injection was available at 60% and experienced the least days of stock out. Magnesium sulfate injection showed more days of both stock out and overstock followed by misoprostol tablet. Proper inventory management practices are significantly influential in ensuring continuous availability of emergency obstetric drugs and may lead to saving lives from PPH and pre-eclampsia.

Several challenges related to medicines inventory management were also identified. If properly addressed, this will ensure proper inventory management practices and thus ensure the availability of medicines. There is also a need for pharmaceutical supply chain management personnel, as they are better trained in medicines management than other healthcare staff.

## Data Availability

The datasets used in this study are available from the corresponding author on a reasonable request.

## Abbreviations

EAC: East Africa community
e-LMIS: Electronic logistics management information system
EmObs: Emergency obstetric drugs
EmOC: Emergency obstetric care
HIV/AIDS: Human immunodeficience virus/ Acquired immunodeficiency syndrome
MCCH: Maternal, child and community health
MSH: Management sciences for health
PE/E: Pre-Eclampsia/Eclampsia
PPH: Postpartum hemorrhage
UNFPA: United Nations population fund
UR: University of Rwanda
WHO: World health organization
